# The Impact of an Early Mobility Protocol on Recovery Outcomes in Patients Undergoing Abdominal Surgeries

**DOI:** 10.7759/cureus.75980

**Published:** 2024-12-18

**Authors:** Binu Xavier, Sasi Vaithilingan, Seethalakshmi Avudaiappan, Panneerselvam Periasamy

**Affiliations:** 1 Department of Medical Surgical Nursing, Vinayaka Missions Research Foundation (Deemed to be University), Salem, IND; 2 Department of Child Health Nursing, Vinayaka Mission's College of Nursing, Puducherry, IND; 3 Department of Nursing Foundation, Sri Ramachandra Institute of Higher Education and Research, Chennai, IND; 4 Department of Physiology, Government Erode Medical College, Perundurai, IND

**Keywords:** early mobility protocol, effectiveness, level of mobility, patient satisfaction, sicu

## Abstract

Background: According to the World Health Organization (WHO), early mobilization is a critical component of healthcare that significantly impacts patient recovery and outcomes. Despite evidence supporting the benefits of early mobilization for abdominal surgery patients, standardized protocols remain scarce across many healthcare environments.

Aim: This study evaluates the feasibility and impact of an early mobility protocol (EMP) on improvement in mobility and patient satisfaction in abdominal surgery patients.

Methods: This feasibility study, conducted in the surgical intensive care unit at Health World Hospital in West Bengal, involved 20 participants who underwent abdominal surgery. Data were collected via demographic questionnaires, the Modified Johns Hopkins Highest Level of Mobility scale, and a patient satisfaction scale. Statistical analysis was performed using IBM Statistical Package for the Social Sciences Statistics, version 28.0, Armonk, NY.

Result: The EMP significantly enhanced recovery outcomes among patients undergoing abdominal surgery. By day 6, six out of 10 participants in the experimental group (EG; 60%) achieved independence in mobility, compared to five out of 10 participants (50%) in the control group. Additionally, the protocol led to substantially higher mobility scores (p = 0.0001) and patient satisfaction levels, with the EG reporting an average satisfaction score of 4.40, markedly higher than the 0.70 observed in the control group (p < 0.05), thus underscoring the protocol's effectiveness.

Conclusion: This study demonstrates that an EMP significantly enhances recovery outcomes for abdominal surgery patients, with 60% (six out of 10) of the EG achieving independence by day 6. However, the lack of standardized early mobilization protocols in healthcare settings remains a critical gap. These results align with the WHO's recommendations, underscoring early mobilization as a cornerstone of postoperative recovery and emphasizing the need for standardized approaches to optimize patient outcomes.

## Introduction

Major abdominal surgeries, vital for treating numerous conditions, come with significant postoperative risks. In 2018, there were 7.4 million such procedures globally, rising to a projected 8.1 million by 2020 from 2010 to 2020. Around 20,000 deaths annually in the United States, Japan, France, Germany, Italy, Spain, and the United Kingdom were linked to complications [1]. Annually, about 310 million major procedures are performed worldwide, including 20 million in Europe and 40-50 million in the United States. These surgeries have a mortality rate of 1%-4%, with 15%-25% of patients experiencing major postoperative morbidity and a similar percentage readmitted within 30 days [1].

Despite advances in surgical techniques, major abdominal surgeries still face significant challenges, including prolonged hospital stays and increased mortality, particularly with gastrointestinal, liver, and pancreatic surgeries [2]. Early mobilization, a key component of enhanced recovery after surgery protocols, helps mitigate these issues by reducing complications, accelerating recovery, improving patient outcomes, and shortening hospital stays, which lowers overall healthcare costs [3].

The need for further research is underscored by a study by Jønsson et al. showing that early intensive mobilization post-high-risk abdominal surgery was well tolerated, with over 80% of patients mobilized at least four times daily by postoperative day (POD) 0 [4]. However, challenges persist for patients with limited mobility, as delayed ambulation continues to significantly impact morbidity and mortality after abdominal surgery [5].

Hu et al. emphasized the need for further research by demonstrating that health education significantly enhances early mobilization in postoperative abdominal patients [6]. Likewise, research from the Kempegowda Institute of Medical Sciences found that early ambulation notably enhanced daily living activities, functional capacity, and psychological well-being. In contrast, prolonged bed rest and delayed ambulation have been shown to impair physical function, emphasizing the need for additional studies to optimize early mobilization strategies [7].

Early postoperative ambulation reduces the risk of venous thromboembolism, improves pulmonary function and circulation, and supports intestinal recovery [8]. Walking after surgery helps maintain breathing patterns, boosts oxygen flow, strengthens muscles, and aids urinary and gastrointestinal function. Conversely, immobility can lead to complications like increased gas, constipation, weakness, higher infection risk, and greater chances of blood clots and lung issues [9].

Early mobilization mitigates the risks of prolonged immobility and surgical stress. Despite advances in surgical techniques, postoperative complications persist, underscoring the need for effective mobilization strategies [10]. This study evaluates the effects of an early mobility protocol (EMP) on patient mobility and satisfaction in a surgical intensive care unit (SICU). The limited research in India on EMP for postoperative abdominal surgery highlights a critical need for further investigation.

## Materials and methods

Study design and setting

This hospital-based feasibility study was conducted in the SICU of Healthworld Hospitals, West Bengal, over a two-month period from October to November 2022.

Study population and sampling

A purposive sampling technique was used to recruit 20 patients who had undergone elective abdominal surgeries, divided equally into control (10 patients) and experimental (10 patients) groups. The study aimed to evaluate the feasibility and preliminary outcomes of implementing the EMP in a clinical setting. It included specific mobilization activities tailored to the patients’ recovery stages. These activities consisted of bed exercises, such as leg lifts and ankle pumps, transitioning to sitting on the edge of the bed, standing, and walking short distances with assistance. The intensity and duration of the exercises were progressively increased based on each patient’s clinical condition and tolerance to ensure safety and efficacy. The limited sample size allowed for assessing protocol adherence and practicality within the constraints of the study period. Participants were proficient in Bengali, Hindi, or English and capable of providing informed consent. Patients with orthopedic issues, bleeding tendencies, or significant comorbidities were excluded.

Inclusion Criteria

The study was designed to ensure a consistent and relevant patient population. Participants aged 18-65 years, scheduled for elective abdominal surgeries, and who were hemodynamically stable were selected. The types of surgeries included in the study encompassed cholecystectomy, hernia repair, and bowel resection. Patients undergoing emergency procedures or those with pre-existing mobility impairments were excluded to maintain the uniformity of the cohort and to accurately evaluate the effectiveness of the EMP. This approach ensured that the results were representative of the target population for elective abdominal surgeries.

Exclusion Criteria

Patients were excluded if they were under 18 or over 65 years of age, had pre-existing mobility impairments, were hemodynamically unstable, or required emergency abdominal surgeries. Additionally, individuals with significant comorbidities such as severe cardiovascular or respiratory conditions, those with contraindications to early mobilization, and patients unwilling to participate were excluded. These criteria ensured the selection of participants for whom the EMP could be safely and effectively evaluated.

Randomization and Group Allocation

Participants were assigned to experimental and control groups through computer-generated randomization, minimizing selection bias. The experimental group (EG) received the EMP in addition to routine care, while the control group adhered to standard nursing care practices.

Baseline characteristics

A comparison of baseline demographic and clinical characteristics confirmed homogeneity between the groups. Both groups included patients undergoing similar types of abdominal surgeries with no significant differences in age, gender, or preoperative health status, ensuring comparability.

Study tools

The study utilized a structured tool comprising three parts (see the Appendix). Part A focused on demographic and baseline characteristics, including age, sex, gender, religion, body mass index (BMI), pain score, and clinical diagnosis. Part B employed the Modified Johns Hopkins Highest Level of Mobility (JH-MHLM) scale to assess mobility levels in bed, chair, and walking [11]. Mobility scoring was categorized into three levels: dependent (1-3), moderately dependent (4-6), and independent (7-8). Part C featured a patient satisfaction scale ranging from 1 to 10, which is designed to evaluate patient-reported satisfaction [11-13]. The tools used in the study were validated by medical and nursing experts, with reliability measured at 0.91.

Data collection

Data collection was conducted using standardized tools at predefined intervals to ensure accuracy and consistency. Trained data collectors adhered strictly to the study protocol, and objective outcome measures, such as mobility scores and patient satisfaction scales, were used to minimize bias despite the lack of assessor blinding. Participants were provided with an information sheet outlining the study's purpose, and written informed consent was obtained to ensure confidentiality. The researcher conducted individual interviews in the SICU and reviewed clinical histories. Pain levels were assessed using the numerical pain rating scale, categorized as mild (1-3), moderate (4-6), and severe (7-10). BMI was calculated using the standard formula: weight in kilograms divided by height in meters squared.

Intervention

The EMP included a series of progressive mobilization activities tailored to the patients' recovery stages. These activities ranged from bed exercises such as leg lifts and ankle pumps to sitting on the edge of the bed, standing, and walking short distances with assistance. The intensity of the exercises was gradually increased based on the patients' tolerance and clinical condition to ensure safety and efficacy. The EMP was administered twice daily to the EG alongside routine nursing care until the POD6. In contrast, the control group received only routine nursing care. Mobility levels were evaluated using the modified JH-MHLM scale on three alternate days up to the POD6, while patient satisfaction was measured before and after the intervention using a 5-point scale ranging from 1 (very satisfied) to 5 (very unsatisfied).

Statistical analysis

Data analysis was conducted using IBM Statistical Package for the Social Sciences Statistics, version 29.0, Armonk, NY. Categorical variables were summarized as frequencies and percentages, and group comparisons were performed using the chi-square test. Changes in mobility over time were analyzed with one-way repeated measures analysis of variance (ANOVA), including post hoc tests for pairwise comparisons. Statistical significance was set at p < 0.05. As this was a pilot study with a limited sample size, the primary aim was to explore trends and assess the feasibility of the EMP. The findings were interpreted cautiously, with a focus on descriptive statistics to provide meaningful context for the outcomes.

Ethical considerations

Ethical approval for the study was obtained from the Institutional Research Committee of IQ City Institute of Nursing Sciences, Durgapur with approval no. IQMC/IEC-10/LTR/10(15)/53 dated November 2, 2022. Participants were informed about the study’s purpose, and written consent was obtained. Data confidentiality was maintained throughout the study, and participation was voluntary.

## Results

Demographic characteristics

The demographic variables of patients admitted to the SICU were compared between the experimental and control groups (n = 20). The chi-square test results indicate no statistically significant differences in age (p = 0.362), gender (p = 0.653), BMI (p = 0.068), education (p = 0.856), religion (p = 0.273), and pain score (p = 1.000). This suggests that the two groups are comparable in terms of these characteristics (Table 1).

**Table 1:** Distribution of demographic variables among patients underwent abdominal surgery in SICU BMI: body mass index; SICU: surgical intensive care unit

Demographic variables	Experimental group, n (%)	Control, n (%)	Chi-square test	p value
Age (in years)				
31-40	-	2 (20)	3.200	0.362
41-50	3 (30)	2 (20)		
51-60	6 (60)	6 (60)		
>60	1 (10)	-		
Gender				
Male	5 (50)	4 (40)	0.202	0.653
Female	5 (50)	6 (60)		
BMI				
25-29.9	4 (40)	8 (80)	-	-
30-34.9	6 (60)	2 (20)		
Education				
High school	5 (50)	4 (40)	-	-
Primary school	3 (30)	3 (30)		
Graduates	2 (20)	3 (30)		
Religion				
Hindu	6 (60)	9 (90)	-	-
Muslim	3 (30)	1 (10)		
Christian	1 (10)	0		
Pain score				
4-6	5 (50)	5 (50)	-	-
7-10	5 (50)	5 (50)		

Our findings show that preinterventional levels of mobility show that both the experimental and control groups had 100% of patients in the "dependent mobility" category, with no patients in the "moderately dependent" or "independent mobility" category. This indicates that before the intervention, all patients were fully dependent on mobility. Table 2 shows that by day 4, significant differences emerged. The EG showed a reduction in "dependent mobility" to 0%, with 90% achieving "moderately dependent mobility" and 10% achieving "independent mobility." In contrast, the control group remained 100% dependent (p < 0.0001). By day 6, 60% of the EG had achieved "independent mobility," while the control group still had 50% in "dependent mobility" (p < 0.004) (Table 2).

**Table 2:** Distribution of level of mobility after intervention among the patient underwent abdominal surgery in SICU SICU: surgical intensive care unit

Time	Level of mobility	Scale score	Experimental group (10), n (%)	Control, n (%)	Chi-square test	p value
Day 2	Dependent mobility	1-3	10 (100)	10 (100)	-	-
	Moderately dependent mobility	4-6	-	-		
	Independent mobility	7-8	-	-		
Day 4	Dependent mobility	1-3	0	10 (100)	20.0	0.0001
	Moderately dependent mobility	4-6	9 (90)	0		
	Independent mobility	7-8	1 (10)	0		
Day 6	Dependent mobility	1-3	0	5 (50)	11.11	0.004
	Moderately dependent mobility	4-6	4 (40)	5 (50)		
	Independent mobility	7-8	6 (60)	0		

Significant improvements were observed in the EG's mobility scores across all postinterventional assessments (p < 0.0001). The control group also showed improvements but to a lesser extent. The interaction effect between assessment time and group was significant (p < 0.05) (Table 3).

**Table 3:** Level of mobility before and after intervention among the patient underwent abdominal surgery in SICU RM ANOVA: repeated measures analysis of variance; SD: standard deviation; SICU: surgical intensive care unit

Group	Preinterventional		Postinterventional-I		Postinterventional-II		Postinterventional-III		One-way RM ANOVA	p value
	Mean	SD	Mean	SD	Mean	SD	Mean	SD		
Experimental group	1.0	0.00	2.70	0.48	5.30	0.82	6.80	1.03	141.0	<0.0001
Control group	1.0	0.00	1.30	0.48	2.30	0.48	3.50	0.52	69.241	<0.0001

It also shows that the two-way ANOVA revealed significant differences between the experimental and control groups in mobility scores (p < 0.0001) (Table 4).

**Table 4:** Summary of statistical results for groups and time points F values and p values for the differences between the groups and over time in the study. Significant at p < 0.05 level

S. no.	Source	F value	p value
1	Between subjects	241.452	0.0001
2	Within subjects	196.766	0.0001

Repeated contrast tests showed significant improvements at all stages for the EG (Table 5).

**Table 5:** Repeated contrast tests: comparison of mobility scores over time between the groups Mobility scores between different time points. It includes the mean differences, F values, and p-values for the overall group and the EG EG: experimental group

S. no.	Comparison	Group	Mean difference	F value	p value
1	Overall	Pretest vs. posttest-I	1.000	90.0	<0.0001
2	Overall	Posttest-I vs. posttest-II	1.800	138.857	<0.0001
3	Overall	Posttest-II vs. posttest-III	1.350	27.224	<0.0001
4	EG	Pretest vs. posttest-I	1.7	40.091	<0.0001
5	EG	Posttest-I vs. posttest-II	2.6	18.581	<0.0001
6	EG	Posttest-II vs. posttest-III	1.5	0.503	<0.496

Intriguingly, no significant associations were found between changes in mobility and demographic variables (age, gender, BMI, education, religion, and pain score) in the EG, indicating that improvements were consistent regardless of these factors (Table 6).

**Table 6:** Association of mean differed score of highest mobility among patients with their selected demographic variables ^*^One-way ANOVA ^**^t test ANOVA: analysis of variance; SD: standard deviation; BMI: body mass index

Demographic variables	Pretest		Posttest		Mean difference		One-way ANOVA/Student independent t test	p value
	Mean	SD	Mean	SD	Mean	SD		
Age (in years)								
41-50	1.00	0.00	6.66	1.52	5.66	1.52	0.037^*^	0.964
51-60	1.00	0.00	6.83	0.98	5.83	0.98		
>60	1.00	0.00	7.00	-	6.00	-		
Gender								
Male	1.00	0.00	6.80	0.83	5.80	0.83	0.000^**^	1.0
Female	1.00	0.00	6.80	1.30	5.80	1.30		
BMI								
25-29.9	1.00	0.00	7.50	1.00	6.50	1.00	1.941^**^	0.104
30-34.9	1.00	0.00	6.33	0.81	5.33	0.81		
Education								
High school	1.00	0.00	6.50	2.12	5.50	2.12	0.165^*^	0.851
Primary school	1.00	0.00	7.00	0.70	6.00	0.70		
Graduates	1.00	0.00	6.66	1.15	5.66	1.15		
Religion								
Hindu	1.00	0.00	6.00	-	5.00	-	1.949^*^	0.212
Muslim	1.00	0.00	6.50	1.04	5.50	1.04		
Christian	1.00	0.00	7.66	0.57	6.66	0.57		
Pain score								
4-6	1.00	0.00	7.20	0.83	6.20	0.83	1.265^**^	0.245
7-10	1.00	0.00	6.40	1.14	5.40	1.14		

Finally, the comparison of satisfaction scores between the groups of patients who underwent abdominal surgery shows that the p value is less than 0.05, indicating that the difference between the groups is statistically significant. The mean satisfaction score for the EG was 4.40 with a standard deviation of 3.10, while the mean satisfaction score for the control group was 0.70 with a standard deviation of 0.99, by which we understand that the EG was more satisfied than the control group.

## Discussion

Our findings demonstrate the efficacy of the EMP in significantly improving recovery outcomes for abdominal surgery patients. Compared to the control group, the EG achieved superior mobility and higher patient satisfaction, with 90% reaching moderately dependent mobility by day 4 and 60% achieving independent mobility by day 6. The EG also reported a mean satisfaction score of 4.40, markedly higher than the 0.70 observed in the control group.

Najjar et al. focused on the relationship between early mobilization and patient satisfaction [12]. It found that patients who were mobilized earlier reported higher levels of satisfaction with their care, corroborating our findings of greater patient satisfaction in the EG. The study highlighted that improved mobility contributes not only to physical recovery but also to psychological well-being, echoing the comprehensive benefits of your protocol. Lower satisfaction levels among some patients could have been influenced by factors beyond perceived delays in recovery and discomfort from immobility. Psychological factors such as anxiety or stress related to the surgery, postoperative pain, and the overall hospital environment, including interactions with healthcare providers and perceived quality of care, may have played a role. Additionally, variations in individual expectations regarding recovery timelines might have impacted satisfaction scores. These factors highlight the multifaceted nature of patient satisfaction and the importance of addressing physical and psychological needs during postoperative care.

Fang et al. conducted a systematic review and meta-analysis to evaluate the impact of early postoperative activities on recovery in patients undergoing abdominal surgery [13]. Their findings highlighted the benefits of EMPs, including faster recovery and improved outcomes. These results align with our study, which demonstrated that the EG receiving early mobilization interventions showed superior mobility scores and higher patient satisfaction than the control group. The analysis of hospital length of stay showed that the EG, which followed the EMP, had a shorter average duration of stay compared to the control group. However, this difference was not statistically significant. These findings suggest a potential trend toward reduced hospitalization with early mobilization, though further studies with larger sample sizes are needed to confirm this observation.

Aldhuhoori et al. conducted a national survey exploring physiotherapy practices for managing patients undergoing upper abdominal surgery in the United Arab Emirates. Their study emphasized the importance of structured physiotherapy interventions in enhancing recovery outcomes [14]. This supports our findings, where implementing an EMP significantly improved independent mobility levels by day 6 postoperatively, demonstrating the efficacy of structured postoperative interventions.

Zang et al. conducted a meta-analysis evaluating the effect of early mobilization in critically ill patients. Their findings revealed that early mobilization significantly reduced hospital length of stay and postoperative complications [15]. This aligns with our study, where the EMP enhanced recovery outcomes and improved patient satisfaction levels, highlighting its critical role in postoperative care.

A prospective study on functional outcomes shows that patients who ambulated early had improved postoperative outcomes, such as reduced length of hospital stay and enhanced mobility, reflecting the progressive increase in mobility scores observed in the EG.

De Almeida et al. conducted a randomized controlled trial demonstrating that an early mobilization program significantly improved functional capacity after major abdominal cancer surgery [16]. Their findings align with our study, which also highlights the importance of structured mobilization protocols in accelerating recovery and enhancing patient satisfaction. Both studies underscore the critical role of systematic early mobility strategies in optimizing postoperative outcomes.

Twomey et al. investigated the impact of early mobilization on recovery after major head and neck surgery with free flap reconstruction [17]. Their study demonstrated that early mobilization significantly improved recovery outcomes and reduced complications. These findings align with our study, where the implementation of an EMP resulted in notable improvements in mobility scores and patient satisfaction, further reinforcing the benefits of early postoperative mobilization.

Limitations

This study had several limitations. The small sample size and single-center design restricted the generalizability of the findings to broader and more diverse healthcare settings. The lack of blinding introduced potential biases, including observer, assessment, and expectation bias, particularly in subjective outcomes such as patient satisfaction scores. Although validated tools and standardized procedures were used to minimize these biases, their influence cannot be entirely ruled out. Additionally, the short follow-up period may not have captured the long-term effects of the EMP. Future multicenter studies with larger sample sizes and longer follow-up periods are needed to validate these findings and assess their broader applicability.

## Conclusions

The findings of this study highlight the critical role of early mobilization protocols in enhancing postoperative care for abdominal surgery patients. Early mobilization was shown to provide specific benefits, including improved functional recovery, reduced dependency, enhanced mobility levels, and increased patient satisfaction. These tangible outcomes underscore the potential of such protocols to optimize surgical recovery and elevate the overall quality of care. Policymakers and healthcare administrators are encouraged to prioritize the integration of early mobilization programs into standard postoperative care, recognizing their evidence-based effectiveness. Standardizing these protocols across healthcare settings could ensure more consistent and favorable patient outcomes.

## Appendices

**Table 7:** Patient information

Patient details	
Name of the patient	-
Hospital registration no.	-
Date of admission	-
Clinical diagnosis	-
Name of surgery	-
Date of surgery	-
Address	-
Comorbidities present	-
Mobile no.	-
WhatsApp no.	-

**Table 8:** Baseline variables instruction: fill or tick (✓) the appropriate options based on patient responses or clinical records BMI: body mass index

S. no.	Base variables	Values
1	Age (in years)	A) 20-30
		B) 31-40
		C) 41-50
		D) >50
2	Gender	A) Male
		B) Female
3	BMI (kg/m²)	A) <18.5
		B) 18.5-24.9
		C) 25-29.9
		D) 30-34.9
		E) ≥35
4	Education level	A) Illiterate
		B) High school
		C) Primary school
		D) Graduate
		E) Postgraduate and above
5	Religion	A) Hindu
		B) Muslim
		C) Christian
		D) Other
6	Pain score (0-10) (Mark on the scale)	⬜ No pain
		⬜ 0
		⬜ 1
		⬜ 2
		⬜ 3
		⬜ 4
		⬜ 5
		⬜ 6
		⬜ 7
		⬜ 8
		⬜ 9
		⬜ 10 (Worst pain)

**Table 9:** Mobility assessment (Johns Hopkins MHLM Scale) instruction: record the mobility level for each postoperative day MHLM: modified highest level of mobility

Mobility level	Score	Day 1	Day 2	Day 3	Day 4	Day 5	Day 6
Walk 250 feet independently	8	-	-	-	-	-	-
Walk 25 feet with one assistant	7	-	-	-	-	-	-
Walk 10 steps with two assistants	6	-	-	-	-	-	-
Stands independently for 1 minute	5	-	-	-	-	-	-
Transfers from bed and sits in a chair	4	-	-	-	-	-	-
Sits at the edge of the bed	3	-	-	-	-	-	-
Attempts to turn self/activity	2	-	-	-	-	-	-
Lying in bed	1	-	-	-	-	-	-

**Table 10:** Patient satisfaction scale instruction to the patient: rate the care received during your stay in the ICU ICU: intensive care unit

Rating	Description
1	Not at all satisfied
2	Not really satisfied
3	Neutral
4	Somewhat satisfied
5	Very much satisfied

**Table 11:** Clinical outcomes and recovery

S. no.	Clinical outcomes	Values
1	Length of hospital stay (days)	-
2	Postoperative complications	A) Venous thromboembolism
		B) Pulmonary issues
		C) Infection
		D) Other
3	Readmission within 30 days (Yes/No)	-

**Table 12:** Scoring procedure

S. no.	Score	Score range
1	Mobility scores	1-3: Dependent mobility
		4-6: Moderately dependent mobility
		7-8: Independent mobility
2	Patient satisfaction	1-2: Dissatisfied
		3: Neutral
		4-5: Satisfied
